# Disturbance and mosquito diversity in the lowland tropical rainforest of central Panama

**DOI:** 10.1038/s41598-017-07476-2

**Published:** 2017-08-03

**Authors:** Jose R. Loaiza, Larissa C. Dutari, Jose R. Rovira, Oris I. Sanjur, Gabriel Z. Laporta, James Pecor, Desmond H. Foley, Gillian Eastwood, Laura D. Kramer, Meghan Radtke, Montira Pongsiri

**Affiliations:** 1 Instituto de Investigaciones Científicas y Servicios de Alta Tecnología, Edificio 219, Clayton, PO, 0843–01103 Ciudad del Saber Republic of Panama; 2 Smithsonian Tropical Research Institute, Panama City, Republic of Panama; 3 Programa Centroamericano de Maestría en Entomología, Universidad de Panama, Panama, Republic of Panama; 4 Centro de Engenharia, Modelagem e Ciências Sociais Aplicadas, Universidade Federal do ABC, Santo Andre, SP Brazil; 5 Setor de Pós-graduação, Pesquisa e Inovação, Faculdade de Medicina do ABC, Santo Andre, SP Brazil; 6 Walter Reed Biosystematics Unit, Smithsonian Institution, Museum Support Center, Suitland, MD United States; 7 Wadsworth Center, New York State Department of Health, Slingerlands, NY United States; 8 US Environment Protection Agency, Washington DC, United States

## Abstract

The Intermediate Disturbance Hypothesis (IDH) is well-known in ecology providing an explanation for the role of disturbance in the coexistence of climax and colonist species. Here, we used the IDH as a framework to describe the role of forest disturbance in shaping the mosquito community structure, and to identify the ecological processes that increase the emergence of vector-borne disease. Mosquitoes were collected in central Panama at immature stages along linear transects in colonising, mixed and climax forest habitats, representing different levels of disturbance. Species were identified taxonomically and classified into functional categories (i.e., colonist, climax, disturbance-generalist, and rare). Using the Huisman-Olff-Fresco multi-model selection approach, IDH testing was done. We did not detect a unimodal relationship between species diversity and forest disturbance expected under the IDH; instead diversity peaked in old-growth forests. Habitat complexity and constraints are two mechanisms proposed to explain this alternative postulate. Moreover, colonist mosquito species were more likely to be involved in or capable of pathogen transmission than climax species. Vector species occurrence decreased notably in undisturbed forest settings. Old-growth forest conservation in tropical rainforests is therefore a highly-recommended solution for preventing new outbreaks of arboviral and parasitic diseases in anthropic environments.

## Introduction

**Figure 1:**
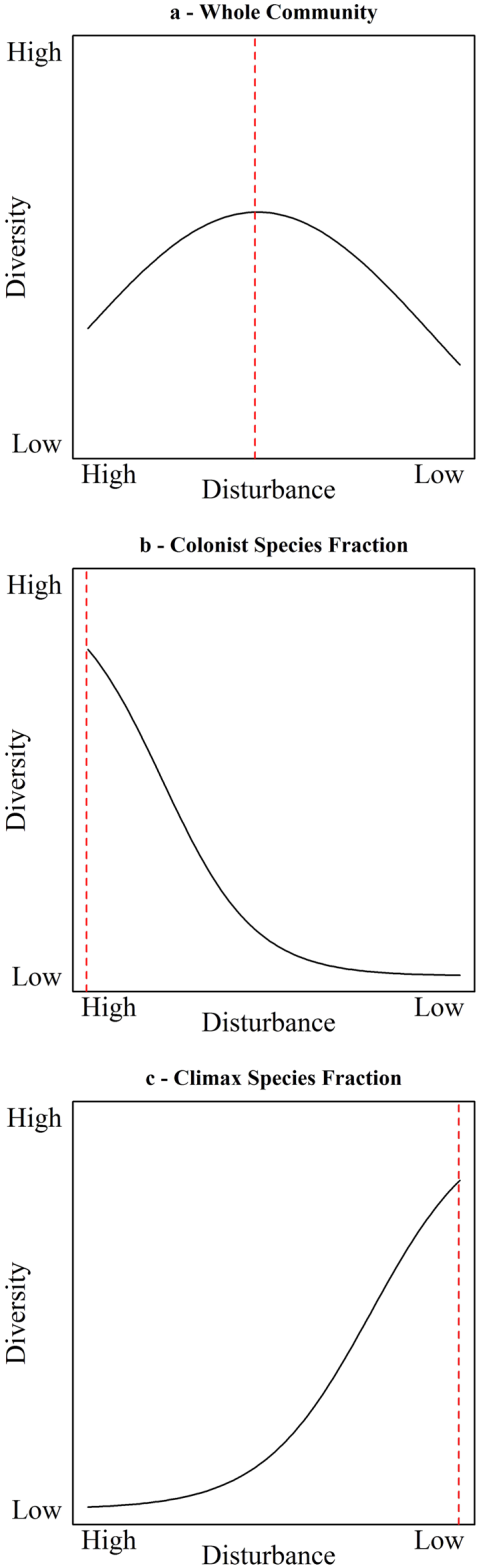
(**a**) The original IDH proposition made by Connell^1^. The author^1^ considered diversity of a given community to be the highest at intermediate disturbance because of co-occurrence of colonist and climax species in the middle of disturbance gradient. From Connell^1^ we derived theoretically that expected diversity of (**b**) Colonist species fraction peaks at high disturbance and that of (**c**) Climax species fraction peaks at low disturbance.

The intermediate disturbance hypothesis (IDH) is one of the most influential and well-known non-equilibrium hypotheses in ecology, providing an explanation for the role of disturbance in the coexistence of climax and colonist species^1^. The IDH postulates that species diversity is highest at intermediate levels of disturbance and declines at low and high levels of disturbance^2^ (Fig. 1a). Following the development of this hypothesis^1^, several studies observed ecological patterns in agreement with the non-equilibrium maintenance of biological diversity^3–8^. This support, coupled with the intuitive nature of the hypothesis, has led to its popularity and acceptance among ecologists^9^. However, recent studies have indicated weak empirical support for and logical flaws in the IDH^10–14^, which has led to substantial debate regarding the scope of IDH and the definitions underlying it^15–17^.

IDH provides an explanation for the non-equilibrium maintenance of biological diversity based on the assumption that, without disturbance, climax or disturbance-intolerant species tend to monopolise resources (e.g., space and food), driving less competitive species to local extinction and reducing overall species diversity^9^. Under high disturbance regimes, species diversity is predicted to be low because only colonist species are able to cope with severe levels of habitat degradation. Connell^1^ found that vegetation in a Ugandan forest followed a successional sequence in which species richness increased during early colonising stages and then declined during late successional stages. This pattern has been referred to as the “narrow IDH definition”^16^. Huston^18^ proposed an extension for this narrow definition and indicated that IDH can also apply more generally to account for disturbance-diversity relationships in non-successional scenarios in tropical rainforests. This definition has been referred to as the “broad IDH definition”^15^. According to this definition, the IDH predicts that disturbance-tolerant species (e.g., colonist) will prevail at high levels of disturbance and that these species will be competitively displaced by disturbance-intolerant species (e.g., climax) under low-disturbance regimes. The IDH also states that these two groups of species should be able to coexist at intermediate levels of disturbance, resulting in a unimodal (‘hump-shaped’) relationship between species diversity and disturbance intensity or frequency (Fig. 1a). Thus, disturbance can act as a reset mechanism whereby populations of competitively dominant species are either periodically subjected to disturbance or subjected to an occasional intense disturbance. As these dominant species are challenged by these disturbance events, critical resources are released for less competitive (‘rare’) species to use^9^. Several empirical evaluations of the IDH have observed this pattern; 46% of IDH-related studies have shown significant species diversity relationships with a unimodal shape^19^. However, the value and utility of the broad IDH definition in ecology is contentious^10–14^. Details about this disagreement can be found elsewhere^14–16^.

Herein, we tested IDH expectations empirically using a species-rich assemblage of Neotropical mosquitoes (Diptera: Culicidae) in order to: (1) describe the role of forest disturbance in shaping the mosquito community structure, and (2) identify the ecological processes that increase the risk of vector-borne disease emergence. We hypothesise that mosquito species diversity will peak at intermediate levels of forest disturbance, following IDH (Fig. 1a). This pattern is expected due to an overlap of optimal habitat conditions for both colonist (Fig. 1b) and climax mosquito species (Fig. 1c) in the middle of the disturbance gradient^1^.

Thenceforth, as an alternative to the IDH proposal, we posit that species diversity will peak at climax forest scenarios, provided that old-growth forest habitats harbour a larger variety of larval habitats plus optimal conditions of water temperature and pH for the interaction of a larger number of species. Next, as a proposition to define the role of forest disturbance on vector-borne disease emergence, we posit that climax and colonist species vary in abundance and somewhat replace each other across a gradient of forest disturbance (e.g., species turn-over), such that within-functional group response to disturbance is more similar than between-functional group response. We also suggest that forest disturbance has a positive effect on the abundance of colonist mosquito species, because it opens opportunities in terms of larval habitat availability and conditions for this group to thrive in altered forest sites. Finally, we link these expectations to the ecological processes that might increase the risk of vector-borne disease emergence, as most colonist mosquito species are likely involved in or are capable of pathogen transmission.

## Results

### The role of forest disturbance in shaping mosquito community structure

**Figure 2:**
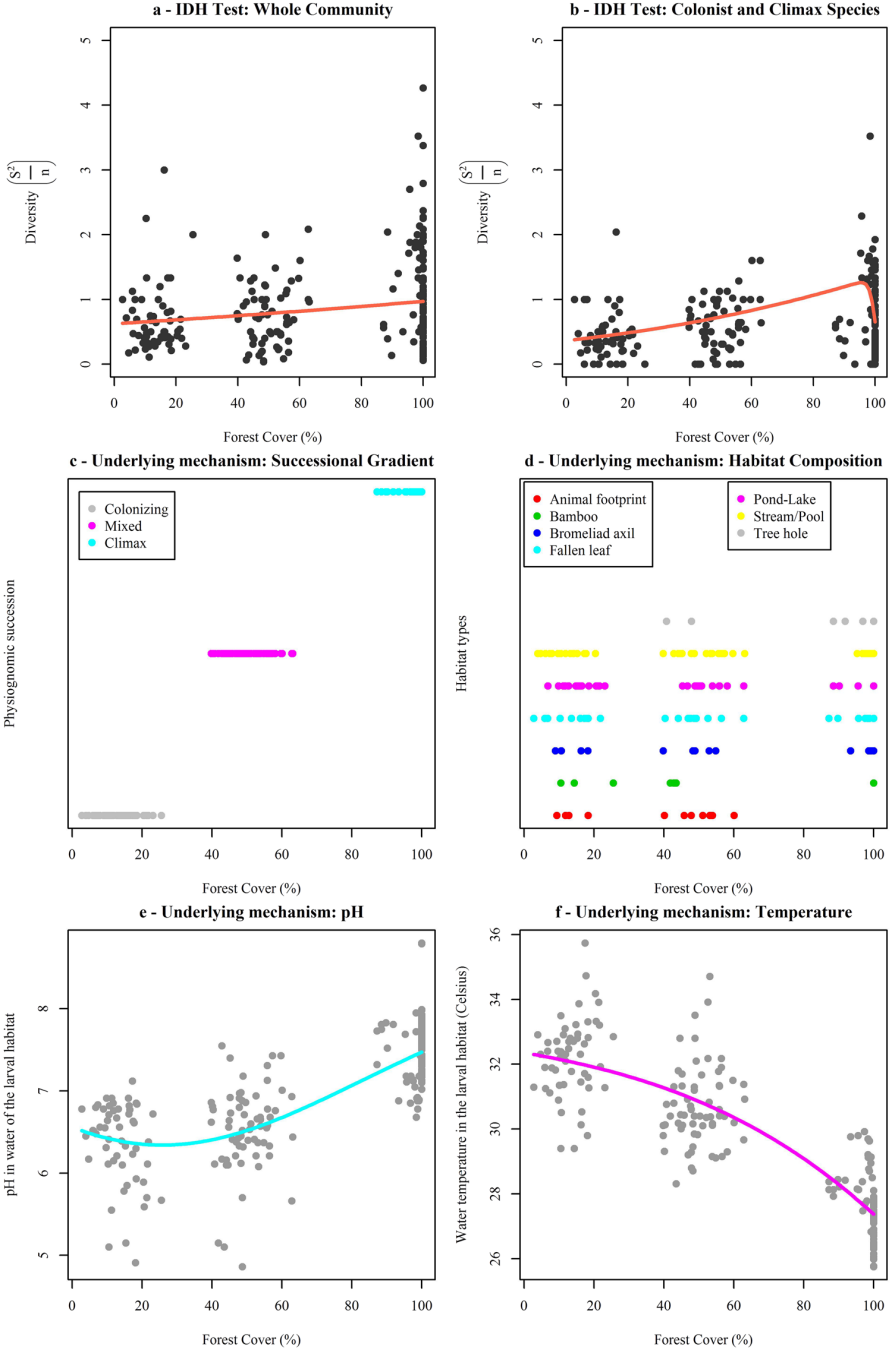
IDH testing. (**a**) whole community. The best-fitting curve was more plausible than the null model (ΔAICc = 1.88; bootstrap likelihood ratio = 4:1) and meant that mosquito diversity increased as forest cover increased and vice-versa. (**b**) colonist and climax species (Connell’s fractions.). The best-fitting curve was slightly more plausible than the null model (ΔAICc = 0.37; bootstrap likelihood ratio = 2.3:1). Underlying mechanisms: 1) habitat complexity, (**c**) successional gradient and (**d**) habitat composition; and 2) larval habitat constraints, **(e)** pH. The best-fitting curve was more plausible than the null model (ΔAICc = 53.2) and **(f)** temperature. The best-fitting curve was more plausible than the null model (ΔAICc = 1,006). R program-scripts and full results of multi-model selection are available upon request.

We empirically tested the assumptions of IDH using 7,839 mosquito larvae belonging to 54 species that were collected from 245 larval sites. These larval habitats were either recipients/containers (natural, artificial) or ground waters. Three categories of forest cover, estimated within a radius of 150-m around each larval habitat, were observed. High forest disturbance occurred in landscape 1 – Las Pavas (2.7–25.5% forest cover), mid-forest disturbance occurred in landscape 2 – Achiote (39.8–63.1% forest cover), and low forest disturbance occurred in landscape 3 – Barro Colorado Island (87.2–100% forest cover), in central Panama. Diversity of mosquito larvae (s^2^/n) varied across different categories of forest cover (%), the latter being used here as a proxy of forest disturbance (Fig. 2a,b). Nonetheless, the mid-disturbance peak (e.g., unimodal ‘hump-shaped’), expected under the IDH, was not observed, either using the whole mosquito community (i.e., colonist, climax, disturbance-generalist, and rare species) (Fig. 2a) or only Connell’s fractions (i.e., colonist & climax species) (Fig. 2b). Therefore, the outcomes of our analysis using mosquito community data from central Panama do not seem to support the assumptions of IDH. Supplementary results about mosquito species classification into functional groups (colonist, climax, disturbance-intolerant, and rare species) can be found in Supplementary Info – Fig. S1.

Mosquito diversity increased as forest cover increased and vice-versa. Potential underlying mechanisms for the observed effect of forest cover on mosquito species diversity are habitat complexity and habitat constraints (Fig. 2c–f).

Habitat complexity is defined here as forest structure (i.e., number of vegetation layers), which increased from low to high forest cover values (Fig. 2c). Furthermore, colonising forest scenarios were mainly composed of herbaceous stratum, whereas mixed forest scenarios were composed of two or more strata, and then climax forest scenarios were represented by old-growth forest with herbaceous, understory, canopy, and emergent layers of vegetation. Larval habitat composition and availability varied along this gradient of vegetation structural complexity (Fig. 2d). For example, the proportion of fallen leaves increased with high forest cover (11 in low, 12 in mid, 20 in high forest cover), as well as ponds (16 in low, 12 in mid, 31 in high forest cover) and stream margins (17 in low, 19 in mid, 32 in high forest cover). Tree holes were only found in habitats of high forest cover (25) and mid-forest cover (2). Animal footprints were observed in low to mid-forest cover habitats (4 and 8, respectively), but none were observed in high forest cover habitats. Four bromeliads were found at low forest cover, 8 at mid-forest cover, and 16 at high forest cover habitats. Bamboo trees were observed equally in all forest cover habitat categories (3 in low, 3 in mid, 2 in high forest cover).

Habitat constraints, water temperature and pH, measured during the time of mosquito collection in each sampling habitat, varied across the gradient of forest cover (Fig. 2e,f). Values of pH increased with forest cover, and for this reason, acidic water was found more frequently in low forest cover larval habitats (Fig. 2e). Water temperature decreased when forest cover increased. For this reason, sunlight-exposed waters were more frequently found in low forest cover sampling habitats (Fig. 2f).

### The ecological processes that might increase the risk of vector-borne disease emergence

We investigated a possible association between mosquito functional classification and vector status using colonist and climax species, which were the most abundant groups in our study. Evidence of vector incrimination for these species in the Republic of Panama or, if not available, in the Neotropical Region are shown in Supplementary info – Table S1. Out of 10 colonists, 8 were considered vectors of important pathogens^20–30^. *Anopheles albimanus, Cq*. *venezuelensis*, *Cx*. *coronator*, *Cx*. *nigripalpus*, *Cx*. *pedroi*, *Ma*. *titillans*, *Ps*. *cingulata*, and *Ps*. *fexox* showed evidence of natural infection in the field and/or high vector competence in the laboratory. Whereas *An*. *albimanus* was mentioned as a main vector for malaria parasites in Central America, the other species were listed as vectors for arboviral pathogens that can infect human and/or domestic animals in that region. *Coquillettidia nigricans* and *Cx*. *declarator* were the only non-vector colonist species of the group. On the other hand, climax species with vector incrimination in the literature were: *Ad*. *squamipennis*, *An. oswaldoi*, *An*. *triannulatus*, and *Li*. *durhamii* ^21, 22, 31–42^. *Aedeomyia squamipennis* was considered a vector of enzootic cycles, as it can transmit avian malaria parasites and the Gamboa virus. Both *Anopheles oswaldoi* and *An*. *triannulatus* were considered auxiliary vectors of *Plasmodium*. A mechanical vector role was considered for *Li*. *durhamii*, as this species could carry eggs of the human botfly.

The other 8 climax species did not have evidence showing that they could vector pathogens to humans or wildlife, so they were classified as non-vectors. We investigated the most recent reports of vector incrimination for the species in the colonist and climax groupings to avoid temporal bias due to past incriminations that do not hold currently. By applying this strategy, we found that 8 out of 10 colonist mosquito species were incriminated as vectors of pathogens to humans or animals, and 4 out of 12 in the climax fraction. A significant effect (*X* ^2^ test statistic = 4.791, p-value = 0.029) for the association between mosquito functional classification (i.e., colonist or climax species) and vector status (i.e., vectors or non-vectors of pathogens) was found (Table 1). This means that the number of vector species was higher in the colonist category whereas more non-vector mosquito species fit into the climax category. Alternatively, our findings could also mean that vector species were more likely to be colonist than climax mosquito species, while the opposite is also true for non-vector species.

**Table 1:** Association of Vector Status (yes, no) with Community Fraction Type (colonist, climax) in a contingency 2by2 table.

	Vector = yes	Vector = no	Total
Colonist	8	2	10
Climax	4	8	12
Total	12	10	22

Odds ratio = 8.00 (95%CI = 1.13, 56.79). *X* ^2^ test statistic = 4.791, p-value = 0.029 (significant result under the null hypothesis: there is no association; significance level (*α*) = 0.05).

## Discussion

### Does mosquito species diversity peak at intermediate levels of forest disturbance (Connell’s IDH model *vs* Fox’s criticism)?

In contrast with several previous studies that found patterns supporting the non-equilibrium maintenance of biological diversity^3–8^, Fox^14^ challenged Connell’s^1^ IDH model, citing a lack of theoretical support. Connell^1^ stated that at intermediate disturbance levels, competitive exclusion declines, allowing for stable coexistence in a non-equilibrium state. Huston^18^ simulated a Lotka-Volterra competition model with mortality events that mimicked the effects of disturbance and supported Connell’s ideas. However, Fox^14^ first cited the lack of evidence that supports competitive exclusion. Fox^14^ also argued that the Huston^18^ model has a subtle outcome. Over the long-term, the increase in average mortality rates cannot produce stable coexistence.

In agreement with the current scientific proposals challenging IDH^11–13^, we did not find a hump-shaped diversity-disturbance relationship using mosquito larvae diversity and forest cover percentage from the lowland tropical rainforest of central Panama. The present test therefore failed to empirically support IDH. Notwithstanding, the main claims of Fox^14^ pointed to logical flaws in the theoretical rationale of IDH: 1) lack of evidence of competitive displacement, 2) not all species have linear responses to disturbance, and 3) increase in average mortality rates in the long-term, are discussed herein, as follows.

The present study found Connell^1^ fractions - colonist and climax species. Colonist mosquito species were associated with low forest covers at one extreme of forest disturbance gradient, whereas climax species were found at the opposite end. This result resembles the mechanism of coexistence of closely related species at the landscape scale (although they can be spatially segregated at the habitat scale), proposed by Laporta and Sallum^43^, after Juliano^44^.

Similarly, it is shown here that colonist *An. albimanus* did not co-occur with two climax species, *An. oswaldoi* and *An. triannulatus*, at the habitat scale (i.e., larval habitat); although the species coexisted at the landscape scale. It is interesting to note further that both *An. oswaldoi* and *An. triannulatus* co-occurred at the larval habitat, but their abundances (n = 278 and n = 138, respectively) were lower than that of *An*. *albimanus* (n = 602). This may indicate that *An. oswaldoi* and *An. triannulatus* interact at the microhabitat scale^43^, so that they need to share resources at the surface of the water on those larval habitats where they co-exist (e.g., ponds and stream margins). The partitioning of resources between *An. oswaldoi* and *An. triannulatus* is likely due to favourable water pH and temperature conditions in shared larval habitats with high forest cover. However, when this high forest cover setting is disturbed, departing from 100% to 20%, it is plausible to assume that both *An. oswaldoi* and *An. triannulatus* (i.e., climax) get displaced^45^ by *An*. *albimanus* (i.e., colonist), an abundant species in colonising disturbed areas^46, 47^. An illustration of the possible competitive displacement of *An*. *albimanus* and *An. oswaldoi*/*An. triannulatus* mediated by disturbance was made herein.

Another group of closely related species is made up by the colonists *Cx*. *coronator*, *Cx*. *declarator*, and *Cx*. *nigripalpus*, with *Cx*. *interrogator*, another climax species of the same subgenus. These *Culex* species also showed co-occurrence at the landscape scale and, potentially, competitive displacement. The competitive displacement could occur when an assemblage dominated by *Cx*. *interrogator* in high forest cover sites is subjected to disturbance and gets replaced by other dominant *Culex* (*Cux*.) species in open areas.

Only 7 species (3 *Anopheles*, 4 *Culex* (*Cux*.)) out of 22 colonist and climax species in our dataset fit with a pattern of competitive displacement. This is partly because semi-permanent water habitats species (i.e., *Coquillettidia*, *Mansonia*, and *Psorophora*) mainly occurred in disturbed areas, whereas tree hole and fallen leaves species (i.e., *Ae*. *terrens*, *An. eiseni*, *Li. asulleptus*, *Li. durhamii*, *Tr. digitatum*) were mostly found in forested sites. For these latter species (e.g., *Ae*. *terrens*), a disturbance event cannot produce another pair of coexisting species, because tree holes were absent in low forest cover. In relation to the first claim of Fox^14^ regarding lack of evidence for competitive displacement, we state that we could find evidence of competitive displacement, but it does not seem to have a large effect.

Fox^14^ also claimed that not all species have linear responses to disturbance. This claim was supported by our outcomes. Highly abundant species in colonist and climax fractions were found. These species had a linear response to the disturbance gradient. For instance, *An*. *albimanus*, *Cx*. *coronator*, *Ps*. *cingulata*, and *Cq*. *nigricans* had peak abundance in the most disturbed areas, and this peak declined towards zero abundance in high forest cover sites. Similarly, *Ad*. *squamipennis*, *Ae*. *terrens*, *An*. *eiseni*, *An*. *trianullatus*, and *Ur*. *geometrica* had very sharp responses on larval habitats with high forest cover, while their abundances were zero at the other extreme. Notwithstanding, there were other species that did not respond linearly to the disturbance gradient, as claimed by Fox^14^. The colonist *Ma*. *titillans* had one peak of high abundance at low forest cover, which is supported by the study of Alfonzo *et al*.^42^, and another with mid-abundance in the middle of the gradient. The climax species *Li*. *durhamii* had two peaks as well, a very high one at high forest cover, supported by Suaza-Vasco^41^, and another with low abundance at intermediate/low forest cover. Additionally, we found five species that can be considered disturbance-generalists, because they had at least two peaks of equal abundances in different forest disturbance categories. This was noticed before for *Cx*. *erraticus* by Alfonzo *et al*.^42^, where they collected 75% of specimens in open areas and 25%, in tall undisturbed forest habitats. Finally, we also found a mid-disturbance species, *Wy*. *simsi*, which had a peak of abundance at the intermediate portion of forest disturbance gradient. The finding of a mid-disturbance species in our dataset is interesting because it means that a community dominated by mid-disturbance species would naturally lead to the expected unimodal diversity-disturbance relationship. Considering specific attributes that enabled species to persist at any given disturbance frequency (e.g., refs 48, 49), our data could support the second claim of Fox^14^.

The third claim of Fox^14^ was that disturbance could increase the average mortality rates in the long-term for rare species. The author simulated from a series of population-based differential equations that species with low abundance (rare) were very likely to become extinct in places exposed to frequent disturbances in the long-term. This claim was supported by our data in full. Twenty-six (48%) of 54 species in our dataset were classified as rare species. Each of these species persists in disturbed areas in a source-sink population dynamics kind of fashion, with Barro Colorado Island as the source of their populations and surrounding areas such as PVAS and ACH as the sink, according to MacArthur and Wilson^50^. Hypothetically, if Barro Colorado Island is exposed to frequent disturbance events, these rare species would probably become locally extinct in the long-term, unless another source for their populations appears.

The fact that we could not empirically support Connell’s^1^ IDH pattern with our dataset is related to the claims of Fox^14^. Competitive displacement did not seem to have a large effect. Species had too many peculiar responses to the disturbance gradient, including a group of disturbance-generalists and one mid-disturbance species. In addition, the high number of rare species indicates that they would not coexist in the long-term in a scenario exposed to constant disturbances, because they would probably become extinct.

### Mosquito species diversity increased towards relatively undisturbed forest environments

We rejected IDH because mosquito species diversity increased with increasing values of forest cover, rather than peaking in the middle of the forest disturbance gradient. Our results depicted an effect of forest cover on mosquito species diversity, meaning that the number of species increased towards healthier and relatively undisturbed forest sites. Although positive and statistically significant, this effect was small. One analogous example of such a small effect is: an increase of 0 to 50% forest cover would increase species diversity by 0.2. However, all 26-rare species (48% of the whole community) were more frequently found in high forest cover.

Underlying mechanisms that could support higher species diversity at high forest cover settings might be habitat complexity and habitat constraints. The former relates to vegetation complexity, which determines the availability and spectrum of different types of larval habitats, whereas the latter is related to chemical composition of the water (i.e., temperature and pH) in those habitats. Vegetation complexity tends to decrease from climax to colonising forest scenarios^51^, thus supporting fewer mosquito species along the disturbance axis due to fewer opportunities to develop and coexist. In contrast, habitat constraints tend to increase in the same direction making it harder for climax and rare species to survive in harsh environments^51^. Fluctuations of habitat complexity and constraints lead to variation in larval habitat properties that are more evident at the opposite extremes of the disturbance gradient.

Moreover, higher habitat complexity in undisrupted forest environments might promote niche differentiation due to a higher variety of larval habitats^51^, while lower habitat constraints in these settings may also favour optimum values of temperatures and pH to yield a larger number of interacting species as larvae in shared aquatic habitats^52^. Our findings further suggest that habitat complexity and constraints are shaped by a successional gradient of forest physiognomic stages, translated into different habitat conditions under three discrete categories of forest cover. Differences in mosquito diversity and abundances across a gradient of forest disturbance could be explained by shifting ecological conditions that affect larval breeding site availability and quality.

### Limitations of the study

The limitations of the present study can be divided into three categories. Ideally, the best study design would match characteristics to cope with these three limitations: (1) The gradient of forest disturbance in this study was not continuous. We had three categories of forest disturbance: a) low forest cover (PVAS), b) medium forest cover (ACH), and c) high forest cover (BCI). In each category, there was a gradient of forest cover, but data were lacking in-between categories. Having more data to fulfil these gaps could help to have a more accurate picture of the effect of forest disturbance on mosquito diversity. (2) Our study design was not a follow-up of successional stages in a single landscape. As our study was a cross-sectional one, the major assumption is that BCI is an undisturbed scenario for ACH (a mid-disturbance scenario) and PVAS (a high-disturbance scenario). During the study design, these areas were selected in the same ecosystem (lowland tropical rainforest), so that they could hypothetically represent the same temporal process of disturbance. Using a cross-sectional study to test a temporal-dependent phenomenon such as the successional stages of Connell’s^1^ IDH is not only challenging, but this was the main critique of Sheil and Burslem^15^, when they replied to the “IDH should be abandoned” by Fox^14^. According to these authors, replacing space-for-time in such a case would not give the same evidence that Connell^1^ found in successional stages of forest in Uganda. This critique made Fox^16^ lower the tone of his statements and agree with the importance of the narrow Connell-based definition of IDH in order to avoid confounding factors. (3) Our study did not experimentally test the effects of forest disturbance on mosquito diversity. It is not trivial to propose an experiment with an intervention such as ‘forest disturbance’. Despite the importance of such an experiment, the opportunities to accomplish this can be very scant. Nevertheless, in places where forest disturbance is out of control, such as in the Brazilian Amazon^53^, one type of intervention could be to compensate landowners for keeping their forest in good shape, and track changes in the mosquito community over time with nearby territories in which landowners would not receive any bonus.

### The ecological process that make risky scenarios for vector-borne disease emergence

While it is true that our results did not provide empirical support for IDH in the field of ecology, using this hypothesis and the criticism around it (i.e., by Fox^14^), as a framework, allowed us to better understand the role of forest disturbance in mosquito-borne disease transmission. Our considerations here also have implications for disease prevention and control. For instance, Lounibos^45^ discussed the applications of competitive displacement as practice for targeting vector population reduction. Our outcomes suggest that competitive displacement could be applied in the Republic of Panama as well as more broadly (i.e., Colombia, Peru, and Central America)^20, 21, 46, 47^ to prevent malaria epidemics. This can be achieved by increasing forest cover recovery in highly disturbed areas (i.e., PVAS and ACH), favouring the likelihood of auxiliary malarial vectors (i.e., *An*. *oswaldoi*/*An*. *triannulatus*)^36, 37^ over primary ones (i.e., *An*. *albimanus*). This control scheme might also be applied to prevent epidemics of arboviral pathogens involving colonists (i.e., *Cx*. *coronator*, *Cx*. *declarator*, and *Cx*. *nigripalpus*) and climax species (i.e., *Cx*. *interrogator*) within the subgenus *Culex* of *Culex*.

In terms of disease transmission^54–56^, focusing on Connell’s fractions (e.g., climax and colonist mosquito species) gave us the opportunity to investigate the role of these assemblages on disease emergence in relation to forest disturbance. Changes in biodiversity caused by recent landscape disturbance in Panama, or in any other place of the tropical rainforest domain^57^, are likely to impact vector species spectra and status over time^58, 59^. We found that the number of mosquito species being incriminated as vectors of pathogens was significantly higher in the colonist fraction than in the climax fraction. Not only was the association colonist–vector species significant, but also it was the most prominent at highly disturbed forest sites, while its likelihood decreased notably towards relatively undisturbed forest settings.

A positive linear response by colonist–vector species to forest disturbance seems to be a tangible process that might increase disease transmission risk in forest-altered tropical areas^58^. ‘*The colonist–vector fraction*’ including the malaria vector (i.e., *Anopheles albimanus*) and zoonotic and bridge vectors of several arboviruses (i.e., *Culex pedroi*, *Culex nigripalpus*, *Psorophora cingulata*, *Psorophora ferox*, *Mansonia titillans*, and *Coquillettidia venezuelensis*) increased in abundance as a function of forest disturbance. According to this view, the so-called spill-over effect might not be a random process, but rather a consequence of forest degradation leading to a higher probability of contact between colonist–vector mosquito species and humans at forest-altered sites. The proportion of different reservoir/hosts existing in forest degraded areas of Panama, will then determine the ultimate disease outcome there, as most mosquito species in forest-altered sites tend to feed opportunistically and upon what is most available nearby. All together, these results suggest a likely role of forest disturbance into vector-borne disease emergence in recently disturbed tropical regions^58, 59^, including Panama.

Nevertheless, considering the well-known suite of entomological studies by Dr. Pedro Galindo in the Republic of Panama^54–56^, just to mention a few, and more recently the theoretical insights by Randolph and Dobson^60^, finding vector species in the colonist and climax fractions should be equally expected. This is theoretically anticipated because female mosquitoes of any particular species have the same opportunities to blood-feed upon vertebrate animals that harbour pathogens. This argument might sound compelling, but it has not been supported by prior studies on vector competence. For instance, Turell by himself^26^ and with colleagues^24, 28, 30^ experimentally tested many mosquito species, thought to be competent transmitters, for infection with different arboviral pathogens, but only partial results could be translated into information on vector incrimination. Cohuet *et al*.^61^ further stated that pathogens could shape vectors.

If it were true that all mosquito species in the community could transmit pathogens equally efficiently, why are those in the colonist fraction of our study more likely to do so? On the one hand, we could rely upon the work of Myers and Patz^58^, who stated that forest disturbance selects the most abundant species, which could lead to higher contact rates with domestic animals and humans, thus increasing the odds of pathogen transmission on these hosts. On the other hand, we should also consider random genetic processes (e.g., neutral effects^62^) as a mechanism that could enhance vector potential at any given time-space. For example, a single mutation event made *Ae*. *albopictus* a superior vector of Chikungunya virus during several outbreaks across Asia and Europe, ten years ago^63^.

**Figure 3:**
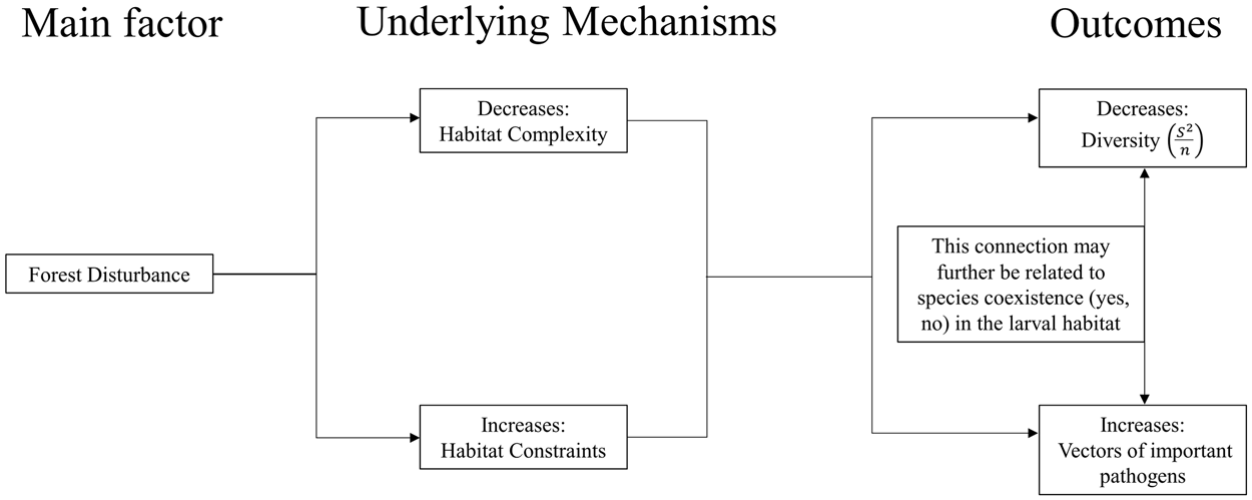
Theoretical synthesis of the results obtained in the present work. The main factor, forest disturbance, decreases habitat complexity and increases habitat constraints. These underlying mechanisms decrease diversity and increase vectors of important pathogens.

Considering the hypothetical synthesis of our work (Fig. 3), a logical statement is that forest disturbance could cause the overall decline of mosquito species diversity, yet at the same time, it could increase disease transmission risk. Although we did not measure disease risk directly, high abundance of colonist–vector species at immature stages was applied here as a proxy for female mosquito population size in forest altered sites. According to this, the deduction would be that one possible mechanism of interconnection of diversity-disease might be related to species interactions in the larval habitat. The authors are conscious of the limitations of the present work, and so the synthetic diagram in Fig. 3 is only intended to guide future research efforts in this direction.

Randolph and Dobson^60^ made a strong critique about the likely effects of high biodiversity as the buffering element to prevent the emergence of vector-borne disease in relatively undisturbed forest habitats. In our study, colonist–vector species such as *An. albimanus* (main vector of malaria in Central America^20, 21, 46, 47^), *Cx*. *nigripalpus* (main vector of St. Louis Encephalitis Virus in the US^26^), and *Cx*. *pedroi* (main vector of the Eastern Equine Encephalitis Virus in Peru^27^) were conspicuous in highly altered forest settings, while their abundances were almost zero at relatively undisturbed forest sites (i.e., old-growth forest). The fundamental conclusion derived from the field of disease ecology is therefore that old-growth forest conservation in tropical rainforests is a reasonable and highly recommended solution for preventing new outbreaks of arboviral and parasitic diseases in anthropic environments.

## Methods

### Study system

**Figure 4:**
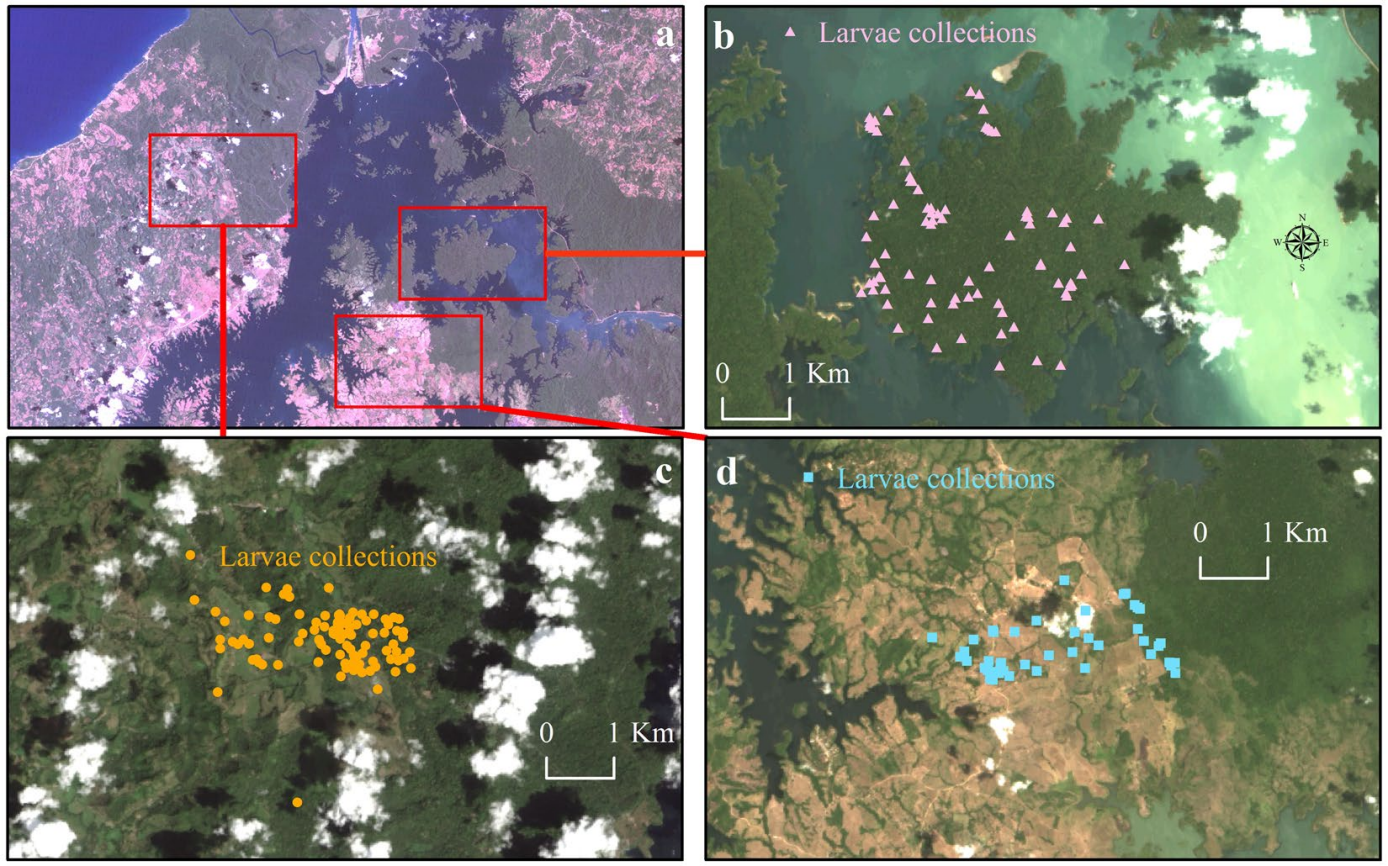
(**a**) The lowland tropical rainforest of Central Panama, 2000. (**b**) The Barro Colorado Island, 2014. (**c**) Achiote, 2016. (**d**) Las Pavas, 2014. Source: Landsat imagery courtesy of NASA Goddard Space Flight Center and U.S. Geological Survey.

This was a cross-sectional and a space-for-time study conducted in the lowland tropical rainforest of central Panama. Herein, we selected three landscapes at different successional stages containing mostly lowland forest and wetland ecosystems^64^ (Fig. 4a). Historically, this area included the Former Panama Canal Zone, a US military territory, with a highly-characterised mosquito fauna due to systematic sampling during the 20^th^ century^65^. Walter Reed Biosystematics Unit (WRBU) Mosquito Catalog^66^ and in-country sources have recorded 286 species of Culicidae in Panama.

Three areas, each expected to having low, medium and high forest disturbances were selected in central Panama for mosquito larvae collection: Barro Colorado Island - BCI (9° 16′ N, - 79° 84′ W; Fig. 4b), Achiote - ACH (9° 22′ N, - 80° 02′ W; Fig. 4c) and Las Pavas - PVAS (9° 09′ N, - 79° 87′ W; Fig. 4d). These areas (herein BCI, ACH, and PVAS) have different climatic conditions due to their geographic position along a Pacific-Atlantic rainfall gradient^67^. BCI and PVAS are positioned closer to the Pacific Ocean; they are drier (1,793 mm of annual rainfall) and more seasonal, experiencing 7 to 8 months of wet season and 4 to 5 months of dry season. In contrast, ACH is situated closer to Isthmus’ Caribbean coast; it is more humid (3,300 mm of annual rainfall) and less seasonal, experiencing year-round precipitation and lacking a well-defined dry season^64, 68^.

Barro Colorado Island (Fig. 4b) is a National Reserve under the custody of the Smithsonian Tropical Research Institute (STRI) since 1923, and is protected by the environmental laws of Panama. The island has a research facility inhabited by a small group of scientists, visitors and administrative personnel, but most of its territory is unoccupied and consists of undisturbed old-growth forest. Hubbell^62^ and colleagues working in BCI proposed the Unified Neutral Theory of Biodiversity, using data collected on plant species abundances.

PVAS and ACH, conversely, are human-altered forest environments that have been gradually colonised during the last 60 years; they have roughly the same number of people, and thus intensity of forest disturbance over these years is thought to be similar. Because of the aforementioned climatic differences between PVAS and ACH, it seems that the latter is more resilient to disturbance because of the higher annual rainfall. This difference can be checked by comparing the satellite imagery taken from the landscape of ACH that shows a later stage of succession (Fig. 4c) in comparison with that observed for PVAS, which resembles an early stage of colonisation (Fig. 4d). Both ACH and PVAS are situated close to a National Park (Fig. 4a); the former borders the Gigante Forest Reserve, whilst the latter borders the San Lorenzo National Park. These landscapes include fragments of old-growth and second-growth forest intermingling with agriculture fields, cattle pastures, and human settlements. The main notion is therefore that they represent a proxy for a hypothetical disturbance scenario of BCI.

### Mosquito sampling and species identification

Immature stages of mosquitoes were collected in linear transects (25 to 30 per each sampling area) of up to 3.5 kilometres (km) long at BCI (mean length 2.88 ± 0.31), PVAS (3.36 ± 0.14) and ACH (3.37 ± 0.14). All mosquito-breeding sites including phytotelmata and ground water sources encountered at 3-m left and right off transects and 2-m of height were checked for the presence of larvae and pupae. Ground water sources were surveyed using a standard larval dipper (350 ml, 13 cm diameter per unit) (BioQuip^®^, Rancho Dominguez, CA). Up to 40 dips (140,000 ml approx.) were taken from each breeding site after ten initial dips to determine positivity. Roots of aquatic vegetation (i.e., mostly *Pistia* and *Eichornia*) in stable breeding sites were rinsed with a 10% solution of Sodium Hydroxide, so that larvae and pupae of *Mansonia* and *Coquillettidia* mosquitoes detached themselves and rose to the surface. Furthermore, Phytotelmata breeding sites were surveyed using a 250 ml pipette and a white plastic tray (25 × 20 × 4 cm) (BioQuip^®^, Rancho Dominguez, CA), with relatively similar amounts of water being extracted as in-ground breeding sites. Samples were placed into Whirl-Pak^®^ plastic bags (118 ml, 8 × 18 cm) (BioQuip^®^, Rancho Dominguez, CA) filled approximately 3/4 full of water from their respective habitats and brought to the laboratory in a cooler container. Later, they were sorted to subfamily level, grouped according to instar in different breeding chambers and link-reared to adult.

Each larval breeding site was photographed with a digital camera and characterised according to environmental features using a standard data collection form^66^. This form included a set of discrete and continuous environmental variables, including degree of shade, water stability, presence or absence of vegetation as well as pH and temperature (°C) of the water, respectively. Water pH and water temperature were measured with a high range HI 98130 Waterproof pH/Conductivity/TDS Tester (Hanna^®^ instruments, Woonsocket, RI). In addition, geographic coordinates (e.g., values of latitude and longitude) for each larval sampling site were recorded using a hand held Global Positioning System (GPS) unit (Garmin^®^ International, Olathe, KS), set to the WGS84 datum and geo-referenced in a Landsat™ 8 OLI satellite image^69^. The bands Red (B4), Green (B3), and Blue (B2) were used to make a RGB composition that was pan-sharpened with the multispectral band (B8) in ArcMAP™ 10.3.1 (ESRI, Redlands, CA), resulting in a 15-m spatial resolution image above which geo-referenced coordinates were represented. This allowed us to estimate the percentage of forest cover within a 150-metre radius around each sampling unit (i.e., larval sampling sites). Sampling transects in PVAS and ACH encompassed areas of low (e.g., >35%), intermediate (e.g., >35% and <65%) and high forest cover (e.g., >65%) in similar proportions, while BCI comprised high forest cover points (e.g., old-growth forest). Mosquito larval collections were carried out every other month from August 2011 to November 2012 in BCI; sampling in PVAS and ACH was conducted in the same fashion from May 2012 to January 2013. A representative portion of the data sets analysed in the present work can be obtained by request from the *VectorMap* portal^70^.

Reared adult mosquito samples were pinned in cardboard triangles, labelled with a unique code/number, and identified using diagnostic morphological characters^71–73^. Species verification was achieved using male genitalia and fourth-instar larval skin preparations following the protocols listed by Thomas Gaffigan and James Pecor^66^. Voucher specimens were deposited at INDICASAT AIP, and also at the University of Panama. In addition, two legs were removed from well-preserved specimens for molecular species confirmation and analysis using the Barcoding region (5′ prime region of the Cytochrome C Oxidase Subunit One mitochondrial gene [*CO1*])^74^. A total of 289 samples for 52 species, initially identified on morphological characters, were randomly taken from the total collected and processed molecularly to rule out potential confounding effects of morphology on the estimation of species diversity. DNA extraction, PCR-amplification and sequencing were done at STRI following standard protocols^75^. We built a neighbour-joining (NJ) tree using *CO1* sequences in MEGA v.5.1^76^ with Kimura 2 parameter (K2P) distances, and bootstrapped the topology with 500 replicates to obtain branch support. We also created a NJ tree using an expanded dataset combining our *CO1* sequences along with additional *CO1* sequences from GenBank^77^ and from The Barcode of Life Data System (bold)^74^.

### Data analysis strategy

The sampling unit in the analysis was the larval habitat or larval sampling sites and for each analysis the sample size used was N = 245, which corresponded to the total number of sample sites from BCI (99), PVAS (46), and ACH (100). The strategy of combining data was three-fold: (1) in relation to the experimental design, which considered a wide range of forest cover values, and then all the sites sampled belonged to a gradient of disturbance, (2) pooling the data resulted in the same outcomes when analysing data from PVAS and ACH separately, and (3) pooled samples from PVAS, ACH and BCI allowed us to increase statistical power of each analysis and to detect deviations from randomness, making possible generalisations about central Panama including pristine and heterogeneous landscapes.

### Mosquito species classification into functional fractions and IDH testing

Each mosquito species in the community was classified as colonist, climax, disturbance-generalist or rare, by employing a multi-model selection analysis for each species’ specific-abundance and forest cover. This resulted in a total of 7 * 54 = 378 models. For the sake of simplicity, we did not show herein all the AICc difference and bootstrap model checking results. Notwithstanding, all these results can be made available upon request.

We first classified mosquito species into distinct functional groups, following Connell^1^. This was done by examining mosquito species-specific abundance response to different levels of forest disturbance, measured by variations in forest cover proportion (%). As a result of this, we divided the mosquito assemblage into colonist (i.e., disturbance-tolerant; Fig. 1b) or climax (i.e., disturbance-intolerant; Fig. 1c) groupings. Furthermore, species that did not respond to disturbance were classified as disturbance-generalists, and species with low abundance (<5% of occurrence) were classified as rare species.

This classification was performed by using the same approach applied by Laporta and Sallum^43^. A multi-model selection scheme was used to fit a specific regression curve to the following data: larval abundance of a given mosquito species gathered from habitats with different proportions of forest cover. Huisman-Olff-Fresco models with extensions compete against each other during this multi-model selection approach (Supplementary Info – Fig. S2)^78^. Model 1 is the null model (the null hypothesis). Models 2–7 are the alternative hypotheses. Model 2 and Model 3 indicate that larval abundance correlates monotonically (i.e., linearly) with forest cover values. Model 4 and Model 5 indicate that larval abundance has a unimodal correlation with forest cover, while Models 6 and 7 indicate that larval abundance has a bimodal correlation with forest cover. The best curve was selected based on a specific set of criteria. The selected model was based on maximum likelihood estimates and the least number of parameters, i.e., the Akaike Information Criteria corrected for small samples (AICc)^78^. Bootstrap model checking was performed to evaluate the robustness of each selected model simulated 100 times^78^.

We tested IDH assumptions by running the same multi-model selection scheme used formerly (Supplementary Info – Fig. S2). This time, the expectation was that estimates of mosquito diversity in response to different levels of forest disturbance would fit the regression curve in Model 4, thus supporting the expected outcome of IDH (i.e., ‘hump-shaped’). First, we tried to fit data from the whole mosquito community to Model 4, and then, we fit data to the same model, but only from the two fractions proposed by Connell^1^. Forest cover was the independent variable and mosquito diversity, the dependent variable. These variables were measured at the scale of the larval habitat, i.e., the micro-habitat scale^43^. In each larval habitat, we estimated the *α*-diversity^79^, herein referred to as diversity. The estimation of diversity followed the procedures adopted by Gotelli and Colwell^80^. The richness of species can increase because of the total abundance, so an adjustment is necessary, named rarefaction^80^. To our data, we applied this idea and then used the squared richness divided by total abundance in each larval habitat.

### Causative mechanisms of ecological and epidemiological outcomes

Underlying mechanisms or possible co-factors that go along with forest disturbance and could therefore be related to the observed pattern of mosquito diversity were also assessed here. These co-factors were: 1) successional gradient of forest types, 2) larval habitat types, 3) water pH and 4) water temperature. All these co-factors were assessed in each larval habitat. Successional gradient of forest and larval habitat types were associated with forest cover variations utilising standard summarising statistics and graphics. Correlations between larval water pH and forest cover and larval water temperature and forest cover were assessed by applying the multi-model (Supplementary Info – Fig. S2) selection approach described earlier.

The vector status of each of the colonist and climax species was investigated by searching the specialised recent literature in Pubmed (5 years or more). We tried, when available, to select evidence in published papers in the last five years to decrease temporal bias. The selection was based on evidence of vector incrimination from either natural infection in field studies or vector competence in laboratory assays. Ecological studies that aimed to understand the vector role in vector-borne disease dynamics were also considered, when the authors discussed the specific role as vector for the studied species. Evidence with a low connection to vector role was not considered. For instance, a species that was positive for arboviral infection in the laboratory, but only one specimen was infected out of 30 tested was not considered a vector. Another species that was found naturally infected with non-infectious stage of a known human pathogen or with the infectious stage of a non-vertebrate pathogen was not considered a vector. An important pathogen was defined herein as any protozoa, microfilaria, and/or arbovirus that could cause clinical infection in a host, either human or animal.

The association between colonist/climax and vector/non-vector was performed by employing a 2 by 2 contingency table. We estimated the odds ratio (OR) with a 95% confidence interval, in which the null hypothesis is OR = 1 (null effect). A *X* ^2^ test was also performed to test the following null hypothesis: H_0_, there is no association. The significance level adopted was 0.05.

## Electronic supplementary material

Supplementary Info

Dataset 1

Dataset 2

Dataset 3
