# Supplementary material for: Disturbance and mosquito diversity in the lowland tropical rainforest of central Panama
Source: Sci Rep. 2017 Aug 3;7:7248. doi: 10.1038/s41598-017-07476-2 (PMC5543164; doi:10.1038/s41598-017-07476-2)
Supplement: Supplementary file 1 — Supplementary Info [file 41598_2017_7476_MOESM1_ESM.pdf]

Disturbance and mosquito diversity in the lowland tropical rainforest of central Panama

Jose R. Loaiza[1,2,3]*, Larissa C. Dutari[1], Jose R. Rovira[1,2], Oris I. Sanjur[2], Gabriel Z. Laporta[4,5]*, James Pecor[6], Desmond H. Foley[6], Gillian Eastwood[7], Laura D. Kramer[7], Meghan Radtke[8] & Montira Pongsiri[8].

Supplementary results

All mosquito species were identified morphologically. A few individuals could be identified only to subgenus, including *Wyeomyia* (*Dendromyia*) sp1, *Culex* (*Melanoconion*) sp1 and sp2, and *Culex* (*Microculex*) sp1 and sp2. DNA barcode sequences were generated from 52 mosquito taxa out of a total of 276 individuals attempted (98% success rate). A few failures were mostly due to the lack of target DNA at the PCR or sequencing steps. All the individuals of morphologically identified mosquito species from central Panama formed lineages with pairwise Kimura-2 parameter (K2P) genetic distances greater than 2%, and could be placed in clusters with high bootstrap support (99%), in agreement with initial morphological designations. All the data used herein were made available (**Supplementary Data S1 online – "The Species Matrix"**, **Supplementary Data S2 online – "The Environmental Data"**, **Supplementary Data S3 online – "The Original Data"**).

Supplementary Data S1 online. The Species Matrix (rows = larval habitat-sampling units, columns = species, cells = number of mosquito larvae collected).

Supplementary Data S2 online. The Environmental Matrix (rows = larval habitat-sampling units, columns = environmental variables, cells = values of each environmental variable).

Supplementary Data S3 online. The Original Data (rows = sampling effort, columns = response and explanatory variables, cells = values of each variable).

Out of a total of 54 mosquito species collected as larvae and pupae in the current work, ten were classified as colonist (**Fig. S1a**) and twelve were classified as climax (**Fig. S1b**). Fitted curves for 9 out of 10 colonist species were model 5 – asymmetrical unimodal, which means that abundance of these species peaked at low forest cover values (**Fig. S1a**). Only *Ma. titillans* in the colonist fraction had as fitted curve model 7 – asymmetrical bimodal, which means that this species was abundant in low forest and moderately abundant in mid-forest cover (**Fig. S1a**). Fitted curves for climax species were from model 7 – asymmetrical bimodal (8 curves, 67%), followed by model 2 – positive linear (3 curves, 25%), and model 4 – symmetrical unimodal (one curve, 8%). These models indicate that species' specific-abundance peaked at high values of forest cover (**Fig. S1b**).

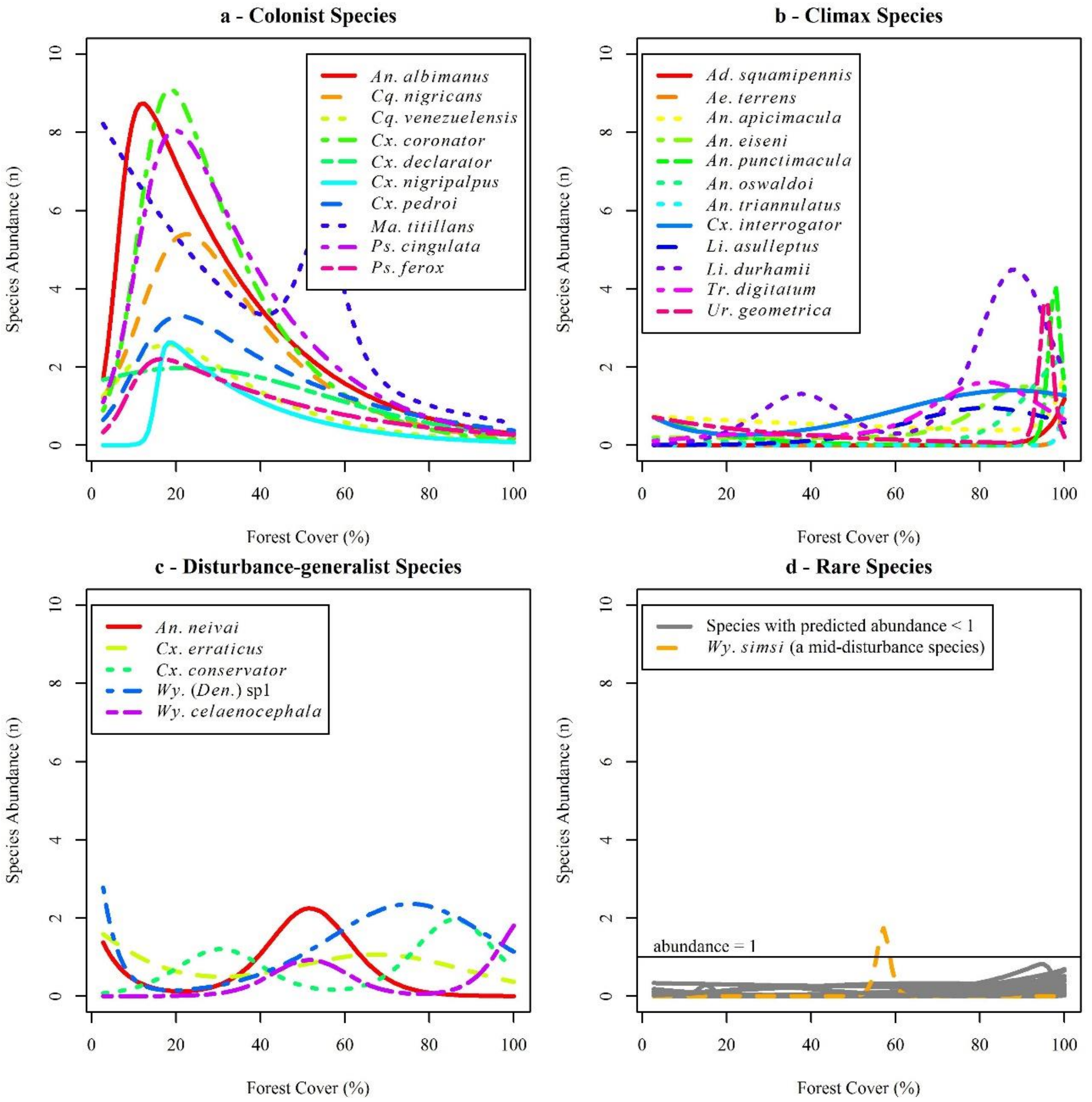

Figure S1. Number of individuals of a species as larvae vs forest cover percentage in each fraction of mosquito community, as follows: (**a**) Colonist Species, (**b**) Climax Species, (**c**) Disturbance-generalist Species, and (**d**) Rare Species. Curves shown in these

plots represent predicted mean abundance according to the best model selected. Selection was based on the Akaike Information Criteria and bootstrap model-robustness checking. R program-scripts and full results of multi-model selection for each species can be available upon request. Models are shown in **Fig. S2**.

Total mosquito abundance for climax species was lower (2,213) than that of colonists (3,754). Per species larvae abundance of colonist mosquitoes was as following: *An. albimanus*=602, *Cq. nigricans*=364, *Cq. venezuelensis*=198, *Cx. coronator*=520, *Cx. declarator*=228, *Cx. nigripalpus*=116, *Cx. pedroi*=292, *Ma. titillans*=698, *Ps. cingulata*=539, and *Ps. fexox*=197. Larval habitats in colonizing forest scenarios yielded 75% of total abundance observed for these species. *Coquillettidia* and *Mansonia* specimens were found most exclusively in ponds and stream margins (99,5%). *Anopheles*, *Culex*, and *Psorophora* specimens were mainly collected in animal footprints (15%), ponds and stream margins (80%). Colonist species were collected most commonly in larval habitats having low water pH (<7) and high water temperatures (29-36°C).

On the other hand, per species larvae abundance of climax mosquitoes was as following: *Ae. squamipennis*=132, *Ae. terrens*=150, *An. apicimacula*=280, *An.eiseni*=165, *An. punctimacula*=218, *An. oswaldoi*=278, *An. triannulatus*=138, *Cx. interrogator*=223, *Li. asulleptus*=95, *Li. durhamii*=320, *Tr. digitatum*=122, and *Ur. geometrica*=92. Ninety percent of the specimens collected from the climax fraction were found in climax forest scenarios. Ninety-nine % of *Ae. squamipennis*, *An. apicimacula*, *An. punctimacula*, *An. oswaldoi*, *An. triannulatus*, and *Ur. geometrica* were found in ponds and stream margins. Specimens of *Cx. interrogator* were found in animal footprints (11%), ponds and stream margins (89%). All *Ae. terrens* specimens were found in tree holes. Eighty-four % of *An. eiseni* specimens were found in tree holes, and 16% in fallen leaves. Ninety-nine % of *Li. asulleptus* specimens occurred in fallen leaves. Specimens of *Li. durhamii* and *Tr. digitatum* occurred mostly in fallen leaves (65%) and tree holes (32%). Climax species were collected most commonly in larval habitats with basic water pH (7-9), and water temperatures ranging between 26 and 30°C.

In addition, five mosquito species were classified as disturbance-generalist: *An. neivai*, *Cx. conservator*, *Cx. erraticus*, *Wy*. (*Den*.) sp1, and *Wy*. *celaenocephala* (**Fig. S1c**). This classification was based on the fitted curves of each species showing two peaks of equal abundance in different categories of forest disturbance. For instance, abundance of *An. neivai* peaked at low and mid-forest cover (**Fig. S1c**). Total abundance of all five species in the current study was 940 and per species abundance was as following: *An.*

neivai=138, *Cx. conservator*=152, *Cx. erraticus*=145, *Wy.* (*Den.*) sp1=247, and *Wy. celaenocephala*=258. There was not any strong association between larval abundance of disturbance-generalist mosquito species and habitat complexity and/or constraints.

Twenty-seven mosquito species were classified as rare (**Fig. S1d**), twenty-six of which were classified in this fraction because their fitted curves were always below the abundance =1 threshold (**Fig. S1d**). Specimens of these 26 species were found mostly in habitats with high forest cover (98%). One rare species had a fitted curve that crossed the abundance =1 threshold, *Wy. simsi*. This species was classified as rare because its response to the forest disturbance was unique. It was recognized as a mid-disturbance species (**Fig. S1d**).

Figure S2. The multi-model selection approach, modified from the study of Jansen and Oksanen[78].

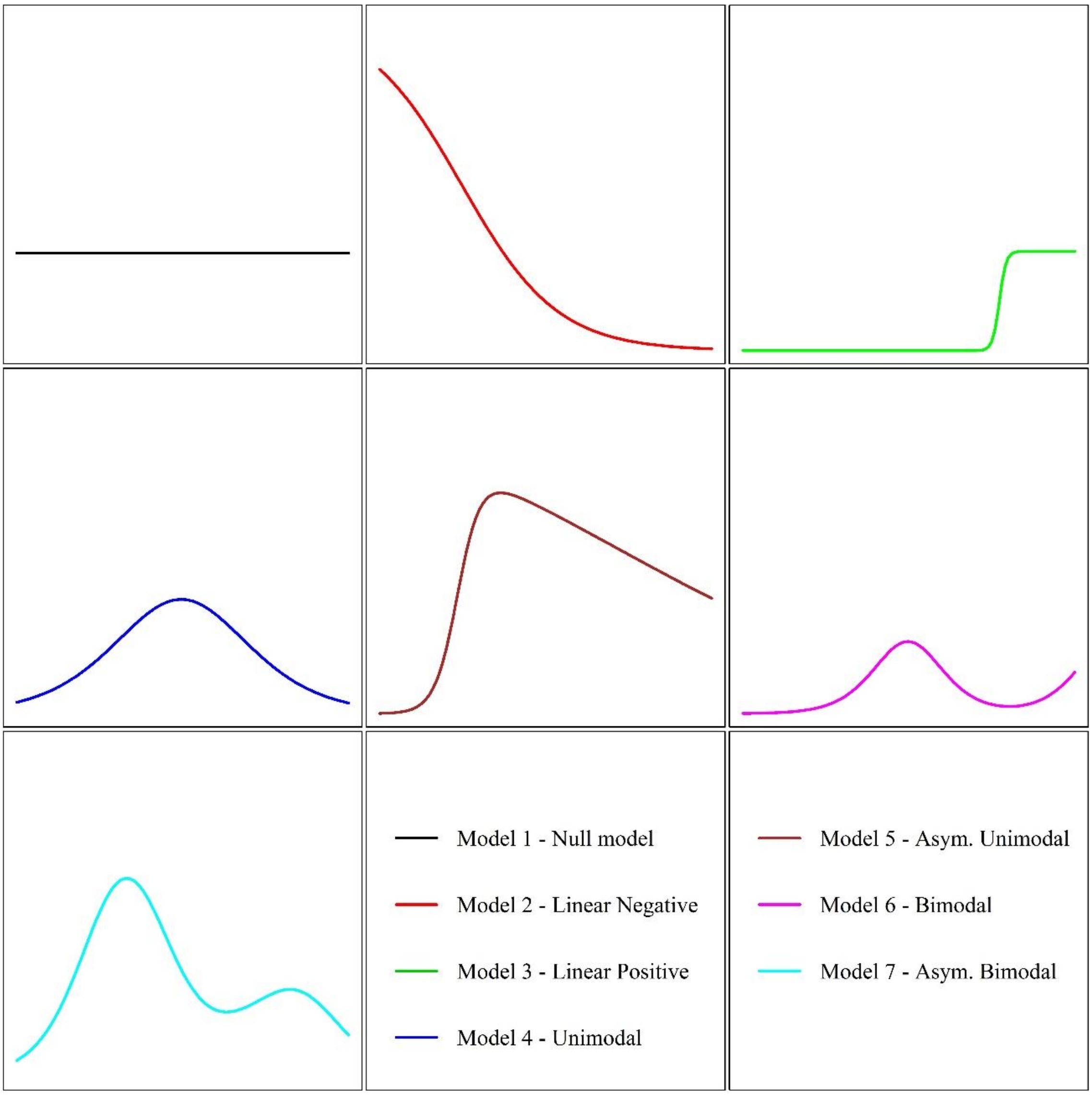

Table S1. Colonist and climax species according to vector status based on evidences of natural infection, experimental transmission, and/or potential vector role of important pathogens in the Latin America region, with emphasis in Panama when available.

Species	Community fraction type	Vector status	Evidence of vector status	Support
An. albimanus	Colonist	Vector	Main malaria-parasite vector in the Pacific coast of Central America, Colombia, and Peru. Found naturally infected with *P. vivax* in a recent outbreak in Panama.	20-21
Cq. nigricans	Colonist	Non-vector	No arboviral isolates in captured specimens in Panama	22
Cq. venezuelensis	Colonist	Vector	Specimens were found naturally infected with Mayaro Virus in Trinidad. This species was susceptible to infection with the Venezuelan Equine Encephalitis Virus (VEEV) in laboratory	23-24
Cx. coronator	Colonist	Vector	It is a competent vector of West Nile Virus under certain parameters in laboratory	25
Cx. declarator	Colonist	Non-vector	One out of 28 individuals were infected with VEEV, and this did not develop a disseminated infection in laboratory	26
Cx. nigripalpus	Colonist	Vector	Main vector of Saint Louis Encephalitis Virus in US.	27
Cx. pedroi	Colonist	Vector	Main vector of the Eastern Equine Encephalitis Virus (EEEV) in Peru	28
Ma. titillans	Colonist	Vector	It was suggested to be an auxiliary vector of filarisis in the British Guiana. This species had a disseminated infection with VEEV in laboratory, suggesting an auxiliary role as vector of this arbovirus	26, 29
Ps. cingulata	Colonist	Vector	This species was susceptible to infection with the VEEV, and had a disseminated infection with the EEEV in laboratory	24, 28
Ps. ferox	Colonist	Vector	Auxiliary vector of EEEV in Peru and a potential vector of Rift Valley Fever Virus in Florida	28, 30
Ad. squamipennis	Climax	Vector	Vector of avian malaria and found naturally infected with Gamboa Virus in Panama	22, 31
Ae. terrens	Climax	Non-vector	A survey of ecology of probable vectors of the Yellow Fever Virus was conducted in Minas Gerais, Brazil, and thus the role of this species as vector was inconclusive	32
An. apicimacula	Climax	Non-vector	Studies conducted to associate Anopheles species with malaria transmission could not succeeded in incriminating this species as vector of malaria parasites in Colombia	33, 34
An. eiseni	Climax	Non-vector	Information is scant on vector role; probably not a vector	35
An. punctimacula	Climax	Non-vector	Although abundant in a survey of probable vectors of a recent malaria outbreak in Panama, no specimen was found infected with malaria parasites	21
An. oswaldoi	Climax	Vector	Found naturally infected with *P. falciparum* in French Guiana	36
An. triannulatus	Climax	Vector	Experimental transmission with *P. vivax* was demonstrated in laboratory	37
Cx. interrogator	Climax	Non-vector	During a survey of vectors of *Dirofilaria immitis* in Mexico, this species was not incriminated as vector	38
Li. asulleptus	Climax	Non-vector	During a survey of probable vectors of VEEV in Venezuela, this species was not considered a vector	39
Li. durhamii	Climax	Vector	Found naturally carrying the human botfly, *Dermatobia hominis*, in the state of São Paulo, Brazil	40
Tr. digitatum	Climax	Non-vector	Abundant species in the Andeans Regions of Colombia, but it is likely not a vector	41
Ur. geometrica	Climax	Non-vector	In an ecology study of probable vectors of VEEV in Venezuela, this species was not considered a vector	42
